# Karyotypic diversification in *Mytilus* mussels (Bivalvia: Mytilidae) inferred from chromosomal mapping of rRNA and histone gene clusters

**DOI:** 10.1186/1471-2156-15-84

**Published:** 2014-07-15

**Authors:** Concepción Pérez-García, Paloma Morán, Juan J Pasantes

**Affiliations:** 1 Dpto. Bioquímica, Xenética e Inmunoloxía, Universidade de Vigo, E-36310 Vigo, Spain; 2 Ifremer, Department of Biogeochemistry and Ecotoxicology, Laboratory of Ecotoxicology, Rue de l’Ile d’Yeu, BP 21105, F-44311 Nantes Cedex 03, France

**Keywords:** *Mytilus*, Chromosome, Fluorescent *in situ* hybridization, Histone genes, Ribosomal RNA genes, Fibre-FISH

## Abstract

**Background:**

Mussels of the genus *Mytilus* present morphologically similar karyotypes that are presumably conserved. The absence of chromosome painting probes in bivalves makes difficult verifying this hypothesis. In this context, we comparatively mapped ribosomal RNA and histone gene families on the chromosomes of *Mytilus edulis, M. galloprovincialis*, *M. trossulus* and *M. californianus* by fluorescent *in situ* hybridization (FISH).

**Results:**

Major rRNA, core and linker histone gene clusters mapped to different chromosome pairs in the four taxa. In contrast, minor rRNA gene clusters showed a different behavior. In all *Mytilus* two of the 5S rDNA clusters mapped to the same chromosome pair and one of them showed overlapping signals with those corresponding to one of the histone H1 gene clusters. The overlapping signals on mitotic chromosomes became a pattern of alternate 5S rRNA and linker histone gene signals on extended chromatin fibers. Additionally, *M. trossulus* showed minor and major rDNA clusters on the same chromosome pair.

**Conclusion:**

The results obtained suggest that at least some of the chromosomes bearing these sequences are orthologous and that chromosomal mapping of rRNA and histone gene clusters could be a good tool to help deciphering some of the many unsolved questions in the systematic classification of Mytilidae.

## Background

The family Mytilidae is constituted by a diverse group of bivalves broadly distributed in marine environments. The systematic classification of Mytilidae (40 genera, 400 species) presents many unsolved problems [1,2]. A good example is the taxonomic status of the mussels belonging to the genus *Mytilus*, an intricate and still not settled subject [3]. Although many different species and/or subspecies of *Mytilus* have been recognized along the years, they can be grouped in two main types. The mussel *M. californianus* Conrad 1835 presents shell ribs and is distributed along the Pacific coast of North America. The mussels belonging to the *M. edulis* complex (*M. edulis* L. 1758, *M. galloprovincialis* Lmk. 1819, *M. trossulus* Gould 1850) are smooth shelled and show a wider distribution range. While *M. trossulus* is confined to northern areas of the Pacific and the Atlantic, and to the Baltic Sea, *M. edulis* and *M. galloprovincialis* have been described, with different specific names, almost worldwide [2-4].

The mussels of the genus *Mytilus* present 2*n* = 28 chromosomes and morphologically conserved karyotypes [5]. Although banding techniques have been applied to the study of their chromosomes [6-9], the correct identification of each chromosome pair in these species is a task far from finished. As in other bivalves [10-12], the accomplishment of that chore requires the use of a broader set of chromosomal markers among which tandemly repeated multigene families are the best candidates.

The nuclear genes for ribosomal RNA in eukaryotes are organized in two multigene families [13]. Major (45S) rDNA is composed of three genes expressing for the 18S, 5.8S and 28S rRNAs separated by two transcribed spacers and an intergenic spacer. Tandem repeats of this unit form clusters at one or more chromosomal pairs constituting the nucleolar organizing regions (NORs). Minor (5S) rDNA repeats consist of a sequence which expresses for the 5S rRNA and a non-transcribed spacer. Clustered tandem repeats of these units also appear at one or more chromosomal pairs. Major rRNA gene clusters have been located by Ag-NOR staining and/or fluorescent in situ hybridization (FISH) in both the species of the *M. edulis* complex and *M. californianus *[14-22]. The location of 5S rDNA clusters has only been reported for *M. edulis* and *M. galloprovincialis *[22].

The genomic organization of the histone genes in eukaryotes shows considerable variation [23]. In the family Mytilidae, the molecular organization of the histone genes has been characterized in *M. edulis *[24,25] and *M. galloprovincialis *[26,27]*.* As in other invertebrate species, histone genes are arranged in clusters repeated in tandem. In *M. edulis* the repeat unit is composed by the four core histone genes (*h4, h2b, h2a, h3*) and is independent of the linker histone genes (*h1*), also repeated in tandem [24,25]. On the other hand, the tandemly repeated unit of histone genes in *M. galloprovincialis* is composed of both core and linker histone genes (*h4, h2b, h2a, h3, h1*) and two 5S rDNA repeats [27]. Linker histone genes also form independent clusters [26]. In *M. galloprovincialis* linker histone gene repeats map to three unidentified chromosome pairs [26] and core histone gene clusters to two, probably coincident with two of the three linker histone gene clusters [27].

Taking into account the above reported differences, we mapped rDNA and histone gene clusters to the chromosomes of *M. edulis*, *M. galloprovincialis, M. trossulus* and *M. californianus* in order to get insights on the chromosome rearrangements that shaped the karyotypes of the species of *Mytilus* and the mechanisms that triggered them.

## Results

**Figure 1:**
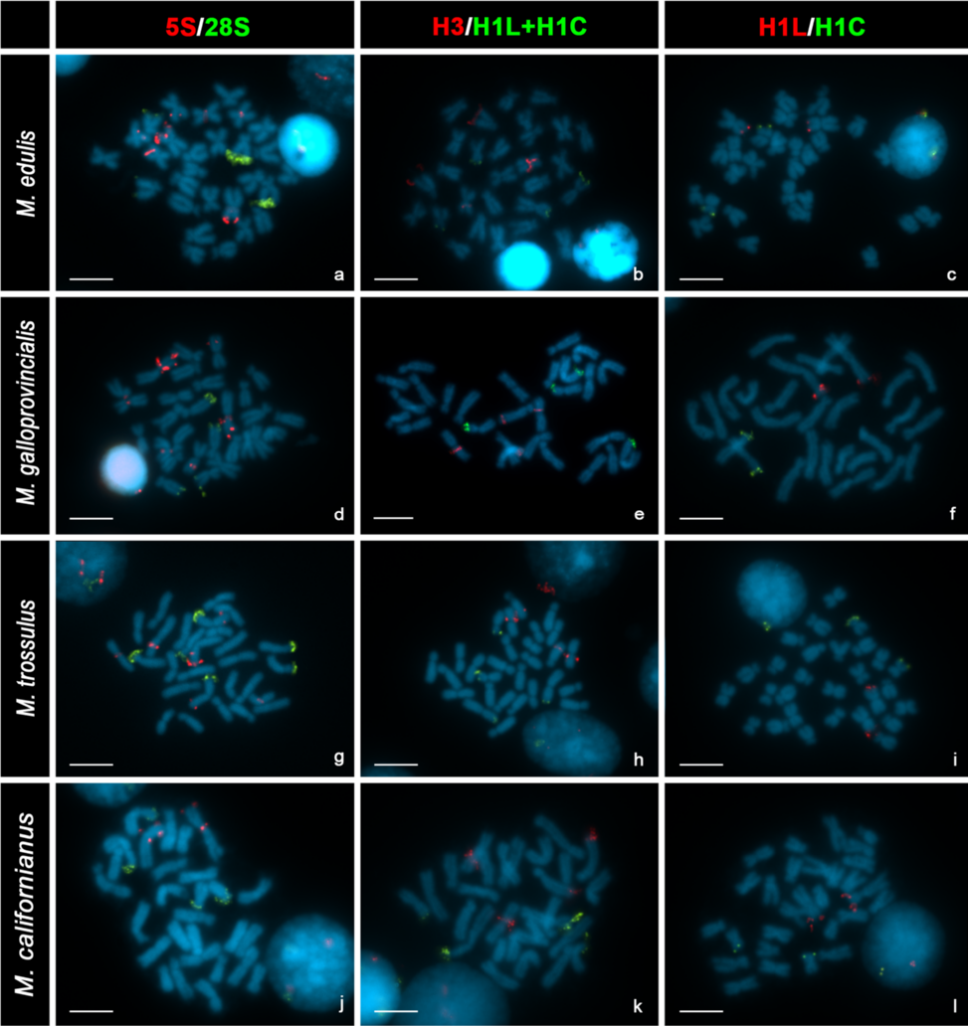
**Mapping of rDNA and histone gene clusters to the chromosomes of four species of *Mytilus *counterstained with DAPI.** Double color FISH experiments using major (28S, green) and minor (5S, red) rDNA probes **(a, d, g, j)** show that major rDNA map to two chromosome pairs in *M. edulis***(a)**, *M. galloprovincialis***(d)** and *M. californianus***(j)** but to three in *M. trossulus***(g)**. All species show two separated 5S rDNA clusters on the biggest chromosome pair **(a, d, g, j)**, however, two additional 5S rDNA loci are present in *M. edulis***(a)**, *M. galloprovincialis***(d)** and *M. trossulus***(g)**. Two color FISH experiments using core (H3, red) and linker (H1L + H1C, green) histone gene probes show signals on four chromosome pairs in all *Mytilus***(b, e, h, k)**; two of the pairs bear signals corresponding to core histone genes and the other two to linker histone genes. Double color FISH experiments using two different linker histone gene probes, one for the *h1* genes linked to the 5S rDNA (H1L, red) and the other for those constituting independent clusters (H1C, green) map to different chromosome pairs in all mussels **(c, f, i, l)**. Scale bars, 5 μm.

**Figure 2:**
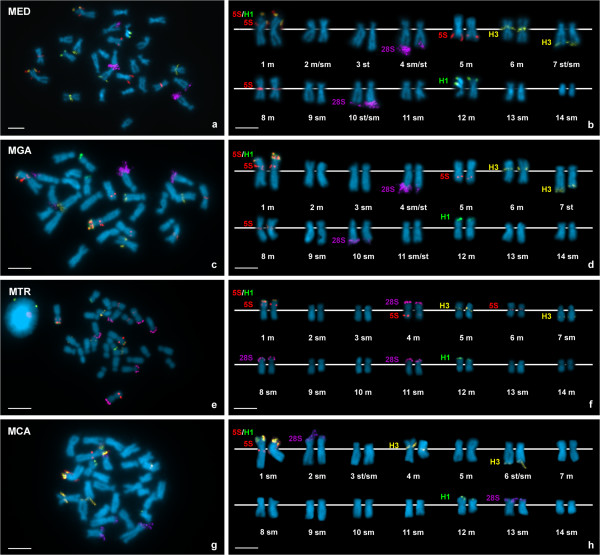
**Chromosomal location of 5S rDNA, major rDNA, core histone genes and linker histone genes in *Mytilus.*** Chromosomal mapping of core histone genes, linker histone genes, 5S rDNA and major rDNA in *Mytilus edulis* (MED; **a**, **b**), *M. galloprovincialis* (MGA; **c**, **d**), *M.trosulus* (MTR; **e**, **f**) and *M. californianus* (MCA; **g**, **h**). Double color FISH using 5S rDNA (5S, red) and linker histone gene (H1L + H1C, green) probes followed by a second double color FISH using core histone gene (H3, yellow) and major rDNA (28S, violet) probes on the same metaphase plates allowed simultaneously mapping these four gene families in *Mytilus*. Chromosomes are arranged in order of decreasing size. Scale bars, 5 μm.

All mussel specimens presented mitotic metaphase plates showing 28 chromosomes (Figures 1 and 2). Representative karyotypes of the four taxa, showing chromosome pairs in decreasing order of size, appear on Figure 2. Relative lengths and centromeric indices are presented in Table 1.

**Table 1:** Relative lengths (RL), centromeric indices (CI) and classification (C) of mussel chromosomes

	***Mytilus edulis***			***Mytilus galloprovincialis***			***Mytilus trossulus***			***Mytilus californianus***		
**Pair**	**RL**	**CI**	**C**	**RL**	**CI**	**C**	**RL**	**CI**	**C**	**RL**	**CI**	**C**
1	9.21 ± 0.29	43.56 ± 0.91	m	9.31 ± 0.35	44.50 ± 1.19	m	9.41 ± 0.23	45.67 ± 0.82	m	9.66 ± 0.40	36.40 ± 0.70	sm
2	8.20 ± 0.25	38.68 ± 0.72	m/sm	8.08 ± 0.17	39.82 ± 0.86	m	8.09 ± 0.24	31.53 ± 1.45	sm	8.52 ± 0.27	34.89 ± 0.95	sm
3	8.05 ± 0.24	20.48 ± 0.90	st	7.91 ± 0.22	27.57 ± 0.91	sm	7.74 ± 0.19	27.95 ± 1.15	sm	8.00 ± 0.25	24.95 ± 0.94	st/sm
4	7.57 ± 0.28	25.35 ± 0.85	sm/st	7.42 ± 0.14	25.96 ± 1.14	sm/st	7.69 ± 0.22	45.48 ± 0.62	m	7.84 ± 0.19	45.63 ± 0.59	m
5	7.41 ± 0.29	45.08 ± 1.07	m	7.26 ± 0.19	46.31 ± 1.16	m	7.43 ± 0.17	45.52 ± 0.75	m	7.76 ± 0.12	41.67 ± 1.10	m
6	7.27 ± 0.19	44.20 ± 0.99	m	7,24 ± 0,18	46.40 ± 0.80	m	7.35 ± 0.27	44.17 ± 0.61	m	7.49 ± 0.16	24.41 ± 1.05	st/sm
7	7.18 ± 0.25	24.33 ± 1.29	st/sm	7.22 ± 0.17	21.71 ± 1.08	st	7.24 ± 0.17	27.22 ± 0.70	sm	7.34 ± 0.14	44.30 ± 1.39	m
8	7.18 ± 0.20	45.19 ± 1.08	m	7.20 ± 0.20	46.14 ± 0.86	m	7.11 ± 0.17	29.61 ± 0.83	sm	7.32 ± 0.22	30.40 ± 1.10	sm
9	6.96 ± 0.21	31.14 ± 1.31	sm	7.01 ± 0.14	31.02 ± 0.95	sm	6.95 ± 0.26	27.81 ± 0.62	sm	6.95 ± 0.21	26.79 ± 1.16	sm
10	6.86 ± 0.20	23.82 ± 1.25	st/sm	6.84 ± 0.22	26.92 ± 1.36	sm	6.81 ± 0.18	41.74 ± 0.83	m	6.72 ± 0.22	34.82 ± 1.20	sm
11	6.39 ± 0.22	26.29 ± 1.37	sm	6.68 ± 0.15	25.47 ± 0.98	sm/st	6.56 ± 0.20	30.76 ± 0.69	sm	6.20 ± 0.21	26.63 ± 0.85	sm
12	6.22 ± 0.20	41.58 ± 1.42	m	6.35 ± 0.20	44.12 ± 0.89	m	6.16 ± 0.13	43.38 ± 1.06	m	5.63 ± 0.23	44.86 ± 0.57	m
13	6.10 ± 0.22	29.98 ± 1.15	sm	5.77 ± 0.19	27.09 ± 0.79	sm	5.88 ± 0.20	33.18 ± 1.11	sm	5.61 ± 0.23	26.73 ± 0.48	sm
14	5.40 ± 0.31	33.13 ± 0.95	sm	5.68 ± 0.15	33.02 ± 0.79	sm	5.59 ± 0.18	42.19 ± 1.15	m	4.97 ± 0.16	31.63 ± 0.89	sm

m: metacentric; sm: submetacentric; st: subtelocentric.

Major rDNAs mapped to two loci in *M. edulis*, *M. galloprovincialis* and *M. californianus* (Figure 1a, d, j) but to three in *M. trossulus* (Figure 1g). The signals were subterminal to the long arms of two submeta/subtelocentric chromosome pairs (4 and 10) in *M. edulis* and *M. galloprovincialis* (Figure 2a-d) and subterminal to the short arms of two submetacentric chromosome pairs (2 and 13) in *M. californianus* (Figure 2h, i). In *M. trossulus* the signals were subterminal to the short arms of metacentric chromosome 4, and submetacentric chromosomes 8 and 11 (Figure 2e, f).

*M. edulis*, *M. galloprovincialis* and *M. trossulus* presented 5S rDNA clusters at four loci (Figure 1a, d, g). As shown in Figure 2a-f, two of these loci were subterminal and intercalary to the short arm of the longest chromosome pair, metacentric chromosome 1, the third was intercalary to the short arm of metacentric chromosome 5 (4 in *M. trossulus*), and the fourth close to the centromere on the short arm of metacentric chromosome 8 (6 in *M. trossulus*). The FISH signals on chromosomes 5 (4 in *M. trossulus*) and 8 (6 in *M. trossulus*) were not always present; intra- and inter-individual variability was detected in the three mussel species. On the other hand, *M. californianus* only presented two 5S rDNA loci (Figure 1j) subterminal to the short arm and intercalary to the long arm of the longest chromosome pair, submetacentric chromosome 1 (Figure 2g, h).

Double-color FISH experiments using major and 5S rDNA probes labeled differently showed that both gene families mapped to different chromosome pairs in *M. edulis, M. galloprovincialis* and *M. californianus*. In contrast, the metacentric chromosome pair 4 of *M. trossulus* bore both major and minor ribosomal gene clusters.

Linker histone gene clusters mapped to two loci in all mussels (Figure 1c, f, i, l). Double-color FISH experiments using two different linker histone gene probes were performed; one of the probes, H1L, was designed to specifically identify the *h1* genes linked to core histone and 5S rRNA genes and the other, H1C, for detecting the *h1* genes clustered independently. The *h1* gene cluster detected with the H1L probe mapped at a subterminal region on the short arm of chromosome pair 1, submetacentric in *M. californianus* and metacentric in the other three species. The second cluster, detected with the H1C probe, was subterminal to the short arm of metacentric chromosome pair 12 in all *Mytilus* species (Figure 2).

Core histone genes also mapped to two loci in the four mussel species (Figure 1b, e, h, k). One of the clusters was close to the centromere on the short arm of metacentric chromosome pair 6 in *M. edulis* and *M. galloprovincialis*, 5 in *M. trossulus*, and 4 in *M. californianus*. The second histone gene cluster was subterminal to the long arm of subtelocentric chromosome pair 7 in *M. edulis* and *M. galloprovincialis* and 6 in *M. californianus* but intercalary to the long arm of sumetacentric chromosome 7 in *M. trossulus* (Figure 2).Double color FISH experiments using core histone (H3) and a mixture of the two linker histone (H1L + H1C) gene probes confirmed that core and linker histone gene clusters mapped to different chromosome pairs in these mussels (Figure 1b, e, h, k).

**Figure 3:**
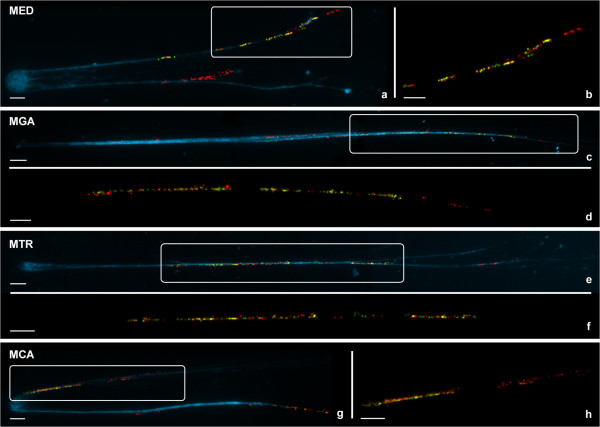
**FISH mapping of 5S rDNA and linker histone genes to release chromatin of *Mytilus.*** Double color FISH experiments using 5S rDNA (red) and H1L linker histone gene (green) probes give patterns of alternant red and green signals on the DAPI-stained chromatin fibers of *M. edulis* (MED, **a**), *M. galloprovincialis* (MGA, **c**), *M. trossulus* (MTR, **e**) and *M. californianus* (MCA, **g**). 5S rDNA clusters devoid of *h1* signals are also present. The pattern of alternant red and green signals, easier to detect on enlarged, unstained chromatin fibers **(b, d, f, h)**, indicates interspersion of 5S rRNA and linker histone genes. Scale bars, 5 μm.

As shown in Figure 2, major rDNA and linker and core histone genes mapped to different chromosome pairs in all *Mytilus* analyzed. In contrast, one of the 5S rDNA and one of the major rDNA clusters mapped to chromosome 4 in *M. trossulus*. Furthermore, the 5S rDNA and *h1 *gene signals subterminal in chromosome 1 overlapped in the four taxa. To determine if the overlapping of the signals was due to interspersion of *h1* and 5S rDNA sequences or just a result of independent clusters not separated enough to give distinct signals, double-color FISH experiments using 5S rDNA and specific chromosome 1 *h1* gene probes were performed on release chromatin fibers. Alternant signals corresponding to 5S rDNA and *h1* gene probes appeared in release chromatin fibers of the four mussel species (Figure 3). 5S rDNA signals devoid of *h1* signals were also detected.

Although the FISH mapping data on Figure 2 showed clear chromosomal differences among mussels, striking similarities were also present. Overlapping 5S rDNA and *h1* gene clusters and a separated 5S rDNA cluster appeared on chromosome 1. A second *h1* gene cluster mapped to a subterminal position on the short arms of a small chromosome (12). Core histone genes mapped near the centromere on the short arms of medium sized meta/submetacentric chromosomes and near the telomere on the long arms of medium sized submeta/subtelocentric chromosomes. On the other hand, the chromosomes bearing major rDNA clusters were different in size and morphology.

## Discussion

### Major ribosomal RNA gene clusters

NORs have been located by silver staining and/or FISH in these species of *Mytilus*. Our results confirm the presence of major rDNA clusters subterminal to the long arms of two chromosome pairs in *M. edulis* and *M. galloprovincialis *[14,15,17,18,22]. In contrast, the occurrence of signals on the short arms of three chromosome pairs in *M. trossulus* and two chromosome pairs in *M. californianus* shows some discrepancies with previous studies. Three NOR-bearing chromosome pairs have been described in *M. californianus *[20,21]; the absence of a major rDNA cluster on the short arms of a third chromosome pair could be attributed, as suggested by González-Tizón et al. [21], to the presence of a low number of copies of major rDNA repeats at this locus. Regarding *M. trossulus*, divergent results for Baltic Sea [15,17] and Eastern Pacific [20,21] populations have been previously reported; the former presenting three clusters at subterminal locations on the long arms of two chromosome pairs and on the short arm of a third pair [15] and the latter showing two clusters at subterminal locations on the short arms of two chromosome pairs and another two at short and long arms of a third pair [20,21]. The divergent location of the NORs has been attributed to the existence of genetic differences between Pacific and Atlantic populations of *M. trossulus *[20] and to the presence of low numbers of major rDNA repeats on the chromosome pair showing two subterminal NORs [21]. Our results displaying only three clusters at subterminal short arms of three chromosome pairs could be explained in the same way.

NORs have been located in another six species of mytilids belonging to the genera *Brachidontes *[28-30], *Perna *[31], *Perumytilus *[32] and *Xenostrobus *[33]. These species show clear differences in the chromosomal distribution of the major rDNA clusters regarding both the number (one to four chromosome pairs) and morphology (metacentric to telocentric) of the NOR-bearing chromosome pairs, and in their chromosomal location (pericentromeric or subterminal to short or long arms), therefore indicating evolutionary changes in these clusters in Mytilidae.

### Minor ribosomal RNA gene clusters

Chromosomal mapping of 5S rDNA clusters has been reported in *M. edulis* and *M. galloprovincialis *[22]. The presence of 5S rDNA repeats clustered at two loci on the same chromosome pair in the four species of *Mytilus* shows that this is the ancestral situation in *Mytilus*. On the other hand, the three species belonging to the *M. edulis* complex present additional 5S rDNA clusters on two chromosome pairs. As reported by Insua et al. [22] for *M. edulis* and *M. galloprovincialis*, the number of FISH signals corresponding to these locations varies from metaphase to metaphase of the same individual. This variation is likely due to the occurrence of a reduced number of copies of the 5S rDNA repeats at those loci, close to the detection limit of the FISH [22]. Intra- and inter-individual variation in the number of repeats [34] together with a low mean number of them, could explain the presence or absence of hybridization signals at those chromosomes. An alternative, although less probable, explanation is that these signals appear as a consequence of independent transposition events that move some of the 5S rDNA sequences to those regions in mussels belonging to the *M. edulis* complex.

The location of 5S rDNA clusters is known in another four species of Mytilidae, *Brachidontes puniceus* and *B. rodriguezi *[30], *Perumytilus purpuratus *[32] and *Xenostrobus securis *[33]. The number of clusters varies from a minimum of two in the species of *Brachidontes* to a maximum of five in *X. securis*. Taking into account that in *P. pupuratus*, *X. securis* and *M. californianus* two of the 5S rDNA clusters map to different arms of a single chromosome pair, this seems to be the ancestral situation in Mytilidae. The presence of the two 5S rDNA clusters on the same chromosome arm of a metacentric chromosome pair in the three species of smooth shelled mussels could be the result of a pericentric inversion on the ancestral submetacentric chromosome still present in *M. californianus*.

In *M. trossulus*, one of the NOR-bearing chromosome pairs also bears a 5S rDNA cluster. Although the occurrence of major and minor rDNA clusters on the same chromosome pair has also been detected in *B. rodriguezi *[30] and *P. purpuratus *[32], the distribution of major and minor rDNA clusters in these species of *Mytilus* suggests that this condition in *M. trossulus* has been probably acquired after the separation from the other smooth shelled mussels, *M. edulis* and *M. galloprovincialis*.

### Histone gene clusters

Core and linker histone gene clusters have been mapped to chromosomes of two species of Mytilidae, *M. galloprovincialis *[26,27] and *X. securis *[33]. FISH mapping of core histone gene clusters has also been performed in another three related mytilids, *B. puniceus*, *B. rodriguezi *[30] and *P. purpuratus *[32]. The detection of separated core and linker histone gene clusters on four chromosome pairs in *Mytilus* coincides with the situation in *X. securis *[33] and fits the molecular findings of Drabent et al. [24] and Albig et al. [25] showing separated linker and core histone gene repeats in *M. edulis* but partially disagrees with FISH mapping data of *M. galloprovincialis *[26,27]. In this species linker histone gene clusters were assigned to three unidentified chromosome pairs [26] and core histone gene clusters to two [27]; the latter were supposed to be coincident with two of the three linker histone gene clusters [27]. The discrepancy of these FISH mapping data with our results showing separate signals for histone genes may be the result of the different composition of the histone H1 gene probes used in the FISH experiments. The PCR generated probes employed in this research included only part of the *h1* coding region, whereas the probe employed by Eirín-López et al. [26] was “an H1 positive recombinant phage from a genomic library”. Therefore, this probe could also contain core histone genes (*h4, h2b, h2a, h3*). If this were the case, the explanation for the divergent results could be straightforward. As proposed by Eirín-López et al. [26], two of their three linker histone gene signals actually correspond to core histone gene clusters, but due to the presence of core histone gene sequences in the probe and not to the occurrence of core and linker histone gene repeats at those loci. The third linker histone gene signal could correspond to one of the two linker histone gene clusters detected by us.

Our histone gene mapping data show a striking conservation on the chromosomal position of these sequences in the four *Mytilus* taxa. Linker histone genes are subterminal to the short arms of the biggest and one of the smallest chromosome pairs. The signals corresponding to probes specific for *h1* genes linked to core histone and 5S rRNA genes appear on the biggest chromosome pair whereas those specific for *h1* gene repeats appear on the smaller one in the four mussels. One of the core histone gene clusters also appears near the centromere on the long arm of a medium sized metacentric chromosome pair in the four taxa. The only mapping difference for histone gene clusters corresponds to the chromosomal position of the second core histone gene cluster, subterminal to the long arm of a medium sized submeta or subtelocentric chromosome pair in *M. edulis*, *M. galloprovincialis* and *M. californianus* but intercalary in *M. trossulus*. The simplest explanation for this condition is the occurrence of a paracentric inversion that transferred the cluster from a subterminal to an intercalary location after the separation from *M. edulis* and *M. galloprovincialis*.

A major difference between *Mytilus* and other mytilids is that in the former core histone gene clusters map to chromosomes not bearing 5S rDNA clusters while in *B. puniceus*, *B. rodriguezi *[30] and *X. securis *[33] one of the histone gene clusters and one of the 5S rDNA clusters are in the same chromosome, either in the same arm, *B. puniceus* and *B. rodriguezi*, or in different ones, *X. securis*. Unlike all the other mytilids, *X. securis* presents four core histone gene clusters instead of two.

### Interspersion of linker histone gene and 5S rDNA clusters

The overlapping *h1* gene and 5S rDNA FISH signals detected at subterminal short arm of chromosome 1 is the result, as demonstrated by fiber-FISH, of interspersion of 5S rDNA and linker histone gene signals. Though molecular data in *M. galloprovincialis* show the existence of gene repeats that include copies of linker and core histone genes (*h4, h2b, h2a, h3, h1*) together with 5S rDNA [27], the FISH signal pattern we found cannot be attributed to the presence of this kind of clusters on that chromosomal position because no core histone gene signals were detected there. Linkage between histone genes and 5S rDNA clusters has been reported in other marine organisms as the crustaceans *Artemia salina *[35] and *Asellus aquaticus *[36]. However, the repeats include both core and linker histone genes in the former and only core histone genes in the latter. Therefore, the interspersed organization of linker histone genes and 5S rDNA described here is the first report of such an association in a marine organism. Given that 5S rRNA and linker histone genes are transcribed by different RNA polymerases, this linkage does not suppose any obvious functional advantage [36] and may represent another example of the reported invasion of different tandemly repeated gene families by 5S rRNA genes through transposition events [37].

The above described results clearly indicate that in these four taxa of *Mytilus*: i) core histone genes are mainly organized in two clusters devoid of linker histone genes [25], ii) linker histone genes appear as tandem repeats, both alone [24,26] and interspersed with 5S rDNA clusters, and iii) the repeats formed by core and linker histone genes (*h4, h2b, h2a, h3, h1*) and two 5S rDNA [27] might be not enough tandemly repeated to be detectable by FISH.

### Chromosome evolution in Mytilidae

The karyotypes of the species of the genus *Mytilus* have been deeply examined [15,18,20,38-43]. All taxa show diploid complements of 2*n* = 28 chromosomes and the karyotypic differences among them are moderately small (Table 1) in comparison with many other groups of organisms. The distribution of rRNA and histone gene clusters also indicates that the chromosome changes accompanying the speciation processes in this genus are relatively low.

Among the smooth shelled mussel taxa that form part of the *M. edulis* complex, *M. edulis* and *M. galloprovincialis* are very close [44] and likely began to diverge about 2 million years ago (MYA) [45]. The karyotypes of these two taxa show a high degree of similitude and the chromosomal distribution of the histone gene and rDNA clusters does not show any differences. The presumably absence of pairing problems in the interspecific hybrids could contribute to the high levels of introgression described in hybrid zones [46]. *M. trossulus* is more distantly related to *M. edulis* and *M. galloprovincialis *[44] and probably started to diverge from the common ancestor of them in the North Pacific 3.5 MYA [45,47]. These divergent life histories are reflected in the differences of their karyotypes and the distribution of the sequences analyzed. In comparison with *M. edulis* and *M. galloprovincialis*, *M. trossulus* presents major rDNA loci on the short arms of three chromosome pairs, major and minor rDNA clusters on the same pair and a more proximal location of one of the core histone gene clusters. These differences can contribute to the reported disruption of gametogenesis in hybrids between these taxa [48]. Estimates about the moment of separation of *M. californianus* vary between 7.6 [49] and 10.7-12.4 MYA [50] and this earlier divergence [44] is also reflected in some morphological differences of their kayotypes and the location of the NORs.

The other four mytilids in which rRNA and histone gene clusters were mapped show somewhat different results. *X. securis* (2n = 30) presents rRNA and histone gene clusters in a total of 11 chromosome pairs [33]. The main differences with respect to *Mytilus* are the pericentromeric location of the NORs and the linker histone gene clusters, the absence of overlapping *h1* and 5S rDNA signals and the presence of a chromosome pair bearing both 5S rDNA and core histone gene clusters. The remaining three species (2*n* = 32), *B. puniceus*, *B. rodriguezi *[30] and *P. purpuratus *[32], show major and minor rRNA and core histone gene clusters in a total of only four chromosome pairs. As in *Mytilus*, major rDNA clusters are subterminal but, in contrast, all core histone gene and most of the 5S rDNA clusters occupy intercalary locations.

## Conclusion

The cytogenetic data presented here indicate that chromosomal mapping of rRNA and histone gene clusters could be a good tool to help deciphering some of the many unsolved questions in the systematic classification of Mytilidae [1,2].

## Methods

### Mussel specimens

Juvenile specimens of *M. edulis, M. galloprovincialis, M. trossulus* and *M. californianus* were collected from intertidal populations at Swansea (Wales, United Kingdom), Baiona (Galicia, Spain), Seattle (Washington, USA) and Santa Barbara (California, USA), respectively. Mussels were maintained in the laboratory in tanks of 5 L of aerated, filtered seawater at 18 ± 1°C and fed on microalgae (*Isochrysis galbana*) for at least 15 days in order to promote both somatic growth and gonadic maturation. The nomenclature used for these taxa follows the World Register of Marine Species database (http://www.marinespecies.org/)

### Mitotic chromosome and release chromatin fiber preparation

Chromosome preparations were obtained following the technique described by Martínez-Expósito et al. [16]. Specimens were exposed to colchicine (0.005%) for 12 h. Gill and mantle tissues were excised and immersed in 50% and 25% seawater for 1 h and fixed with ethanol/acetic acid for 1 h. Chromosome spreads were obtained by dissociating small pieces of tissue in 60% acetic acid and dropping the cellular suspension onto slides heated to 50°C.

Chromatin fibers were released according to Fidlerovà et al. [51]. Cellular suspensions were centrifuged for 10 min at 1200 rpm and the pellet was re-suspended in fixative and dropped onto slides. After leaving to evaporate for a short time, slides were immersed in 1x PBS for 1–2 min and the chromatin fiber was released with NaOH (0.05 M in 30% ethanol).

### DNA extraction, PCR amplification and probe labeling

Total DNA was extracted following the method of Estoup et al. [52] with minor modifications. Approximately 3 mg of adductor muscle tissue was homogenized in 0.4 ml of a pre-warmed (60°C) 10% Chelex 100 (BioRad) solution. After adding pronase (1.4 mg mL^-1^) and incubating for 1 h at 60°C in agitation, the extracted DNA was stored at 4°C.

FISH probes were obtained by polymerase chain reaction (PCR). Amplifications were performed in 20 μl of a solution containing 50 ng DNA, 1x PCR buffer, 0.5 mM each dNTP, 2.5 mM MgCl_2_, 1 μM each primer and 1 U BIOTAQ DNA polymerase (Bioline).

**Figure 4:**
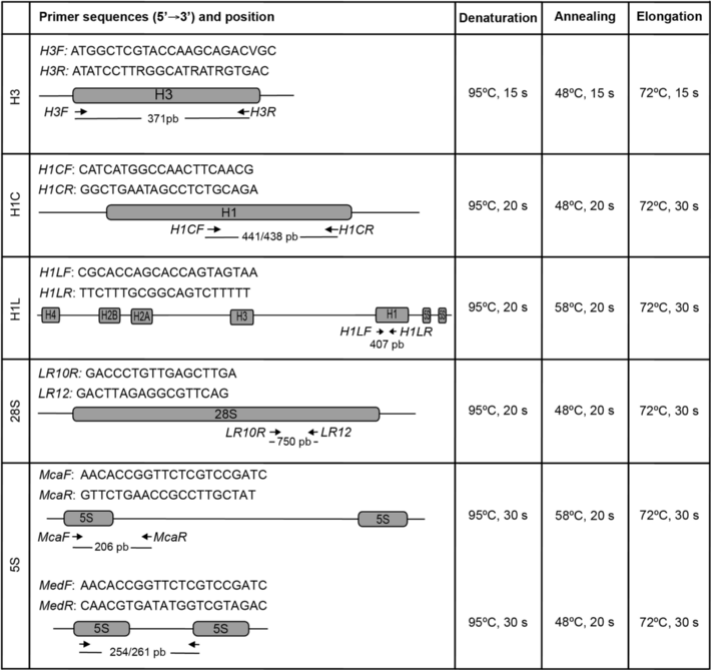
**Primers and parameters used in the PCR.** Schematic representation of the amplified regions that includes the primers sequences, the region of annealing and the sizes of the amplified products. PCR parameters are also included.

The primers employed in the amplifications appear in Figure 4. Universal primers retrieved from the Vilgalys Lab website (R. Vilgalys, Duke University, Durham, NC [http://www.biology.duke.edu/fungi/mycolab/primers.htm]) were used to amplify a fragment of the 28S rRNA gene of the major rDNA repeat (*LR10R, LR12*). The amplification of the 5S rDNA in *M. californianus* was performed using primers (*McaF* and *McaR*) designed from its 5S rDNA sequence [53]. For the other three species of mussels the primers employed for the amplification of the minor rDNA (*MedF, MedR*) were designed from the sequence of the 5S rRNA of *M. edulis *[54]. Two different sets of primers were designed to amplify linker histone genes. The first set (*H1LF* and *H1LR*) was intended to amplify the *h1* genes linked to core histone genes and 5S rDNA clusters described in *M. galloprovincialis *[27]. The second (*H1CF* and *H1CR*), designed after aligning the nucleotide sequences of the linker histone genes from *M. edulis*, *M. galloprovincialis*, *M. chilensis*, *M. californianus* and *M. trossulus*, was intended to amplify those clustered independently [24,26]. The amplification of the H3 histone genes was performed using primers described by Giribet and Distel [55].After 5 min denaturation at 95°C, 30 cycles of amplification were performed using the conditions shown in Figure 4. A final extension step of 7 min at 72°C was applied. All reactions were carried out in a GeneAmp PCR system 9700 (Applied Biosystems) and PCR products were examined by electrophoresis on 2% agarose gels. Single products were obtained after amplification using each set of primers. 28S rDNA probes were labeled with biotin-16-dUTP (Roche Applied Science) and/or digoxigenin-11-dUTP (10× DIG Labeling Mix, Roche Applied Science) using a nick translation kit (Roche Applied Science). Linker histone gene, histone H3 gene and 5S rDNA probes were directly labeled by PCR either with biotin-16-dUTP (20 μM) or digoxigenin-11-dUTP (5 μM). The labeled PCR products were precipitated before FISH.

### Fluorescent *in situ* hybridization (FISH)

Single and double FISH experiments were performed following methods previously published [33]. Preparations were denatured at 69°C for 2 min and hybridized overnight at 37°C. Signal detection was performed using fluorescein avidin and biotinylated anti-avidin for the biotinylated probes and mouse antidigoxigenin, goat anti-mouse rhodamine and rabbit anti-goat rhodamine for the digoxigenin-labeled probes. Slides were counterstained with DAPI (4′,6-diamidino-2-phenylindole; 0.14 μg mL^-1^) and mounted in antifade (Vectashield, Vector). In order to map four probes on the same metaphase plates, two sequential FISH experiments were performed. The probes employed in the first hybridization were biotin-labeled linker histone genes and digoxigenin-labeled 5S rDNAs. After visualization and photography, the preparations were re-hybridized using biotin-labeled core histone gene probes and digoxigenin-labeled major rDNA probes and the same metaphase plates were examined and photographed again.

Slide visualization and photography were carried out using a Nikon Eclipse-800 microscope equipped with an epifluorescence system. Chromosome counting and karyotype analysis were performed in 40 specimens, 10 per species (5 males, 5 females). A minimum of 5 individuals per species and 20 complete metaphase plates per individual were recorded for each probe or combination of probes. Separated images for each fluorochrome were obtained using a DS-Qi1Mc CCD camera (Nikon) controlled by the NIS-Elements software (Nikon). The merging of the images was done with Adobe Photoshop.

For each species, 10 complete metaphase plates showing FISH signals were used to construct karyotypes. Chromosome and arm lengths were carefully measured and relative lengths and centromeric indices were calculated. Chromosome nomenclature follows Levan et al. [56].

## Abbreviations

DAPI: 4′,6-diamidino-2-phenylindole; FISH: Fluorescence *in situ* hybridization; MCA: *Mytilus californianus*; MED: *Mytilus edulis*; MGA: *Mytilus galloprovincialis*; MTR: *Mytilus trossulus*; MYA: Million years ago; NOR: Nucleolus organizing region; PCR: Polymerase chain reaction; rDNA: Ribosomal DNA; rRNA: Ribosomal RNA.

## Competing interests

The authors declare that they have no competing interests.

## Authors’ contributions

CPG did most part of the cytogenetic procedures and collaborated on the molecular work, the bibliographic review, and the writing of this paper. PM participated in developing the molecular techniques and helped in the writing. JJP coordinated the study, helped in developing the laboratory techniques and cytogenetic analyses and coordinated the writing of the manuscript. All authors read and approved the final manuscript.
